# Understanding Crowd-Powered Search Groups: A Social Network Perspective

**DOI:** 10.1371/journal.pone.0039749

**Published:** 2012-06-27

**Authors:** Qingpeng Zhang, Fei-Yue Wang, Daniel Zeng, Tao Wang

**Affiliations:** 1 The State Key Laboratory of Management and Control for Complex Systems, Chinese Academy of Sciences, Beijing, China; 2 Department of Systems and Industrial Engineering, The University of Arizona, Tucson, Arizona, United States of America; 3 Department of Management Information Systems, The University of Arizona, Tucson, Arizona, United States of America; 4 The Research Center for Computational Experiments and Parallel Systems, National University of Defense Technology, Changsha, Hunan, China; Universidad Carlos III de Madrid, Spain

## Abstract

**Background:**

Crowd-powered search is a new form of search and problem solving scheme that involves collaboration among a potentially large number of voluntary Web users. Human flesh search (HFS), a particular form of crowd-powered search originated in China, has seen tremendous growth since its inception in 2001. HFS presents a valuable test-bed for scientists to validate existing and new theories in social computing, sociology, behavioral sciences, and so forth.

**Methodology:**

In this research, we construct an aggregated HFS group, consisting of the participants and their relationships in a comprehensive set of identified HFS episodes. We study the topological properties and the evolution of the aggregated network and different sub-groups in the network. We also identify the key HFS participants according to a variety of measures.

**Conclusions:**

We found that, as compared with other online social networks, HFS participant network shares the power-law degree distribution and small-world property, but with a looser and more distributed organizational structure, leading to the diversity, decentralization, and independence of HFS participants. In addition, the HFS group has been becoming increasingly decentralized. The comparisons of different HFS sub-groups reveal that HFS participants collaborated more often when they conducted the searches in local platforms or the searches requiring a certain level of professional knowledge background. On the contrary, HFS participants did not collaborate much when they performed the search task in national platforms or the searches with general topics that did not require specific information and learning. We also observed that the key HFS information contributors, carriers, and transmitters came from different groups of HFS participants.

## Introduction

In the past five years, *human flesh search* (HFS) has become an explosive Web phenomenon. The term, “human flesh,” is translated from its Chinese root and refers to human empowerment. In previous studies, HFS was formally defined as a Web-facilitated crowd behavior aimed at accomplishing a goal-oriented task of common interest through the online sharing and disseminating information acquired from both online and offline sources [1], [2]. As a form of “*crowd-powered*” search, HFS shares many common characteristics with crowdsourcing [3], [4] and the emerging social search engines [1], [5]. Since its debut in 2001, HFS has been widespread and drawn a lot of attention after a series of public and successful searches against animal abuses and false pictures in 2006–2007. Since then, the frequency of HFS episodes has risen drastically [1].

Currently, HFS has been widely used as a common public medium for Web users to find the people’s identity and information, as well as the causes and truth of events. In order to be successful, HFS participants from one or more online communities collaborate with each other across various web platforms. The types of episodes range from a series of social desirable episodes (anti-corruption, anti-animal abuses, public safety, traffic hit and run, etc.) to social undesired episodes (inappropriate exposure, Net mobs, etc.) and neutral episodes (mystery good-looking people, rumors concerning celebrities, etc.) [6]. HFS has revealed certain very interesting and unique collaboration and crowd mobilization patterns, which are occurring every day on the Web. Since data of the Internet-associated mobility of crowds is mostly accessible to the public, HFS presents a valuable test-bed for scientists to validate existing and new theories in social computing, sociology, behavioral sciences, etc. From a network science point of view, the HFS group is a vast dynamic evolutionary network, with massive human collaboration among groups of voluntary Web users sharing a common goal [1], [2], [6]. From a sociology perspective, HFS activities could be considered as a type of cyber-enabled social movement organizations. Moreover, the empirical data of HFS, open in the Web [6], can lead to new theoretical developments in psychology, social and political sciences. Various other research topics could be raised from studying and modeling HFS phenomena. However, due to the difficulty of defining and identifying HFS episodes, rigorous research on understanding HFS is still lacking and much needed.

Researchers have employed social network analysis to study the evolution and structure of a wide variety of online groups and communities, including blogsphere [7], [8], [9], [10], [11], Twitter [12], [13], online forums [14], [15], social networking sites [16], [17], [18], movie and user comments [19], and so forth. After successfully unveiling the scale-free and small-world properties [8], [20], scientists were able to model and predict human behaviors based on the analysis from the rich web data [21], [22]. In 2010, Wang et al. presented the first empirical study of HFS and studied the topology features of HFS networks of two typical episodes [1]. Their results suggested that HFS shared many common features of other online groups and communities, but possess very unique characteristics, including its uniquely rich online/offline interactions, star-like topology, and information synchronization through a small number of efficient knowledge transmitters [1]. Based on these findings, Zhang et al proposed an SBA model to interpret the star-like topology of HFS participant network [23]. Another modeling approach has been introduced to incorporate network expansion and propagation with feedback [24]. In addition to the effort of modeling HFS, a recent study of Japanese HFS episodes tried to explain the motivation behind HFS from the aspect of expectancy theory and information prospectability [25].

**Figure 1:**
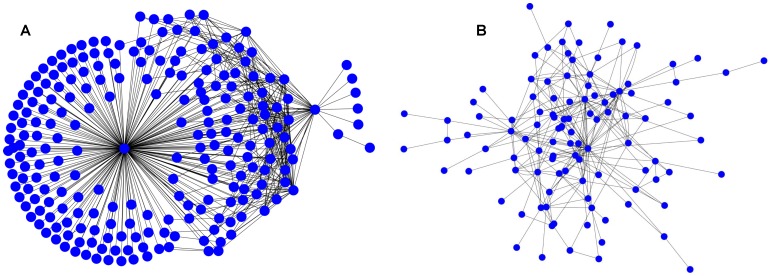
A typical HFS participant network. (A) with casual nodes, and (B) without casual nodes.

Although several works on HFS have been conducted, existing studies have mainly focused on case studies and network modeling from intuition [1], [6], [23], [24], [25]. Especially, it is unclear how the collaboration patterns involve and vary from different taxonomic groups and different platforms. Without a comprehensive understanding of the HFS group, as what has been accomplished in understanding blogospheres, researchers could not build realistic models to capture the real characteristics of HFS and develop applications based on similar crowd behaviors. Therefore, a comprehensive and detailed study of the HFS group is necessary to support and boost future research.

In this study, we attempt to address a series of questions that could shed light on the true understanding of the HFS phenomenon: (a) How does the network topology of the HFS group differ from other online social networks? (b) What characteristics that the HFS group possesses are important for the success of search tasks? (c) How does the HFS group evolve in terms of its network structure? (d) What are the differences in collaboration patterns on different platforms; especially do the co-location and expertise concentration associated with the platforms matter for the collaboration patterns of the HFS group? (e) What are the differences in collaboration patterns of different types of HFS sub-communities? (d) Do the key information contributors, key information carriers, and key information transmitters come from the same groups of participants in the HFS community?

**Figure 2:**
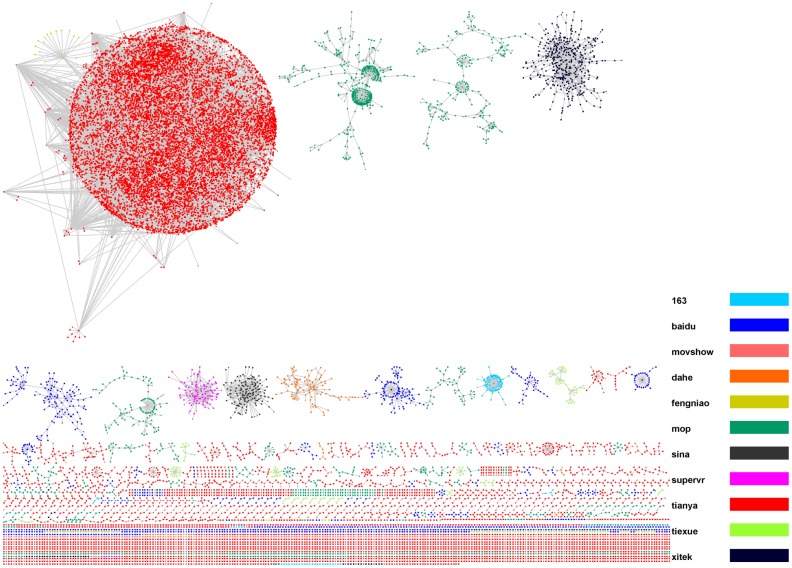
The HFS group network visualization. The color of a node represents the platform where the node belongs to.

The organization of this paper is as follows. The Results and Discussion section presents the main body of our work. We first introduce the dataset and the data retrieval method in Data subsection. Then we use social network analysis to unveil the topological properties of an aggregated HFS community and compare it with other online communities in The HFS as One Network section. In the end of this section, we identify the key HFS participants according to different measures and look into the distribution of the key information contributors, carriers, and transmitters. The subsections of Comparison of Different Platforms and Comparison of Different Types of HFS Episodes reveal and discuss two interesting facts that co-location and expertise concentration lead to more collaboration in HFS behaviors, which are different from the scientific collaboration characteristics observed by previous research. Finally, we conclude the paper with remarks for future work in Conclusion section.

**Table 1:** Platforms for HFS.

Platform	Description
*163*	Web portal for news comments and forums
*baidu*	Web portal for searching, forums, blogs, and web service
*dahe*	Forum for local discussion
*fengniao*	Forum for photography enthusiasts
*movshow*	Forum for pet enthusiasts
*mop*	Forum for general discussion nationwide
*sina*	Web portal for news comments and forums
*supervr*	Forum for pet enthusiasts
*tianya*	Forum for general discussion nationwide
*tiexue*	Forum for military fans
*xitek*	Forum for photography enthusiasts

## Materials and Methods

Currently all existing studies on HFS were based on individual case studies [1], [6], [23], [24], [25] since there is no clear cut to define what a typical HFS community is. Researchers studying blogosphere have used blogs from one or more servers to represent the blogosphere [7], [8], [9], [26]. Works on coauthorship and citation network have employed datasets provided by digital libraries like ISI Web of Science, IEEE Explore, ACM Digital Library, JSTOR, and so forth [27], [28], [29], [30]. Studies on Twitters have built micro-blogging communities by monitoring the public timeline for a period or using a set of keywords and key users for data collection [12], [13]. For this research, we have collected the most comprehensive dataset of HFS discussion threads of online forums and news comments from typical HFS episodes during the past decade (2001–2010). To ensure the correctness and comprehensiveness of the dataset, we have employed both manual and automatic detection, identification, and information collection of HFS episodes by human experts and computer programs [1], [6]. In order to better reflect the HFS collaboration patterns revealed so far, here we have built an aggregated HFS network to represent the entire HFS group using the information of all the participants who had collaborated with others and the citation/reply-to relationship among them for the period from 2001 to 2010.

**Table 2:** The topological properties of the HFS group.

Measure	HFS Group
*N*	20813
*L*	29798
Δ	0.0001
*NC*	2821
*N_G_* (%)	11556 (55.5%)
*<d>*	2.650
*C*	0.027
*l*	8.679
*D*	28
*λ_in_*	2.1
*λ_out_*	2.4
*r*	0.127
*r_in_*	0.054
*r_out_*	0.191

*N*: number of nodes; *L*: number of links; Δ: network density; *NC*: number of components; *NG*: number of nodes in the giant component; *<d>*: average degree; *C*: average clustering coefficient; *l*: average shortest path length; *D*: network diameter; *λ_in_*: power of in-degree distribution; *λ_out_*: power of in-degree distribution; *r*: total degree assortativity coefficient; *r_in_*: in-degree assortativity coefficient; *r_out_*: out-degree assortativity coefficient.

The data collection involves identifying HFS episodes manually (via browsing through the Web), and searching news media for second-hand reporting and comments about HFS episodes both manually and automatically [1], [6]. After a particular HFS episode was identified, we first gained an in-depth understanding of its context, initiation, progression, and outcomes by going through both first-hand (e.g., postings on forums or video-sharing sites with a large number of followers) and second-hand materials (e.g., media reports) manually. We then used a Web crawler to systematically collect information from past online posts including participants' online ids, these participants' IP addresses (if shown online), the full text of these posts, and the timings of replies. This allowed us to categorize the development of the behaviors and to explore the actions, both online and offline, taken by the groups involved. At present, we have identified a set of 487 HFS episodes from its inception in 2001 through November 3, 2010. For all those episodes, we have collected the basic information including the name, starting and ending date, type, estimated population size of participants involved, final result, etc. Analysis based on the basic information has been reported in our previous works [1], [6]. Since many old episodes were no longer accessible on the Internet, we were only able to collect the original discussion threads of 200 episodes. Furthermore, we excluded those episodes without citation/reply-to relationship among participants. In the end, the dataset used in this study contains 98 HFS episodes with 904,823 posts generated by 397,583 distinct users in our dataset.

**Table 3:** Bow-tie structural comparison of HFS group and other online communities.

	SCC	IN	OUT	TENDRIL	TUBE	DISC
Web [32]	0.277	0.212	0.212	0.215	0.004	0.080
Wikipediacommunity [34]	0.824	0.066	0.067	0.006	0.0002	0.037
Question &answering community[14]	0.123	0.549	0.130	0.175	0.004	0.019
Blogosphere [53]	0.239	0.568	0.103	N/A	N/A	N/A
Twitter community [54]	0.080	N/A	N/A	N/A	N/A	N/A
HFS Group	0.096	0.105	0.135	0.213	0.007	0.444

**Figure 3:**
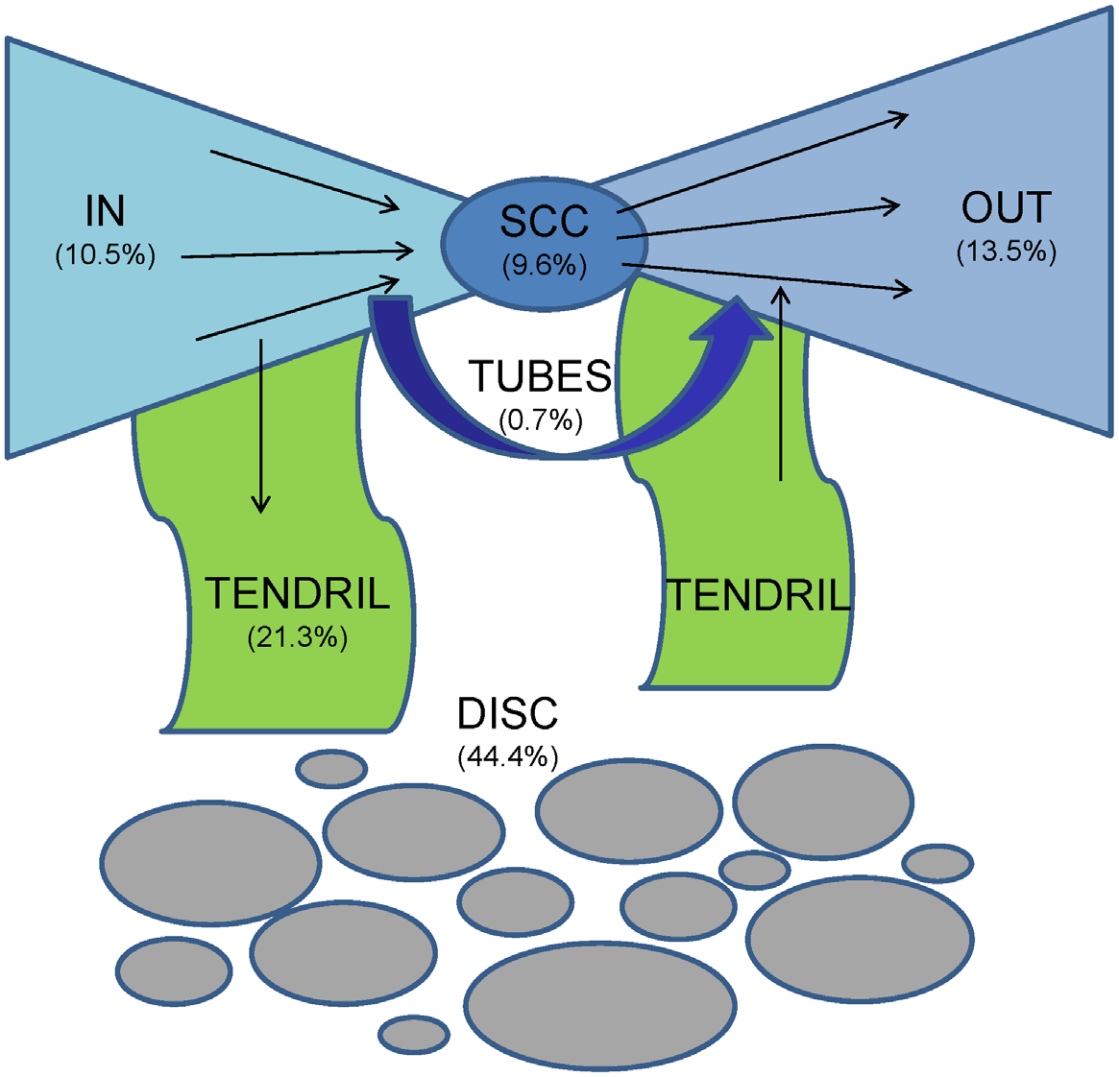
The bow-tie structure of the HFS group.

We constructed HFS participant networks using the cross-citation/reply-to relationship. In an HFS participant network, each node is corresponding to a unique user ID, which is usually associated with one distinct HFS participant. The edges between pairs of nodes indicate the presence of Web posting citations between them [1], [2], [6]. In our previous works, we focused more on the information propagation, thus linked all follow-up nodes to the initial node for each discussion thread [1]. As a result, the networks had a star-like topology, indicating a broadcast pattern (see Figure 1 for visualization). However, 94.8% nodes in the HFS networks that we collected only linked to initial nodes, and no citations were related to them due to the nature of online forum discussion. We denoted this type of nodes as casual nodes and the corresponding participants as casual participants. The existence of large portion of casual nodes is due to the fact that HFS groups are the cyber-enabled inclusive movement organizations (as compared to the exclusive movement organizations)–since the requirement to participate HFS is low, a large number of Web users were able to join HFS groups easily, but only a small fraction of them collaborated for conducting actual searches [31]. Although casual nodes helped spread HFS information and keep discussion threads in the spotlight on different online forums (most online forums displayed discussion threads by the time of last reply posted in descending order), those nodes did not contribute to the actual collaboration activities during HFS.

In this study we were only interested in how HFS participants collaborated with each other as unveiled by the citation/reply-to relationship. Therefore, we excluded casual nodes and analyzed the remaining aggregated HFS participant network, as shown in Figure 2, which involved a total of 20,813 distinct nodes and 29,798 distinct edges from 2005 to 2010.

**Figure 4:**
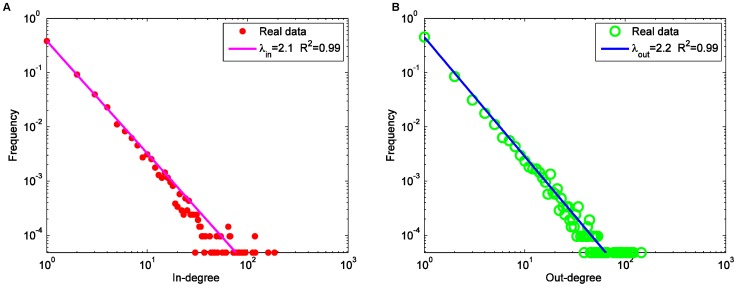
The degree distributions of the HFS group: (A) in-degree; (B) out-degree.

**Table 4:** Comparison of the HFS group and other online communities.

Type of Online Communities	Observed Characteristics
HFS group	Power-law degree distribution (λ_in_ = 2.07, λ_in_ = 2.20), power-law activity distribution (λ_being cited_ = 1.75, λ_citing others_ = 1.84), loose organization, small-world, bow-tie structure with a large portion of TENDRIL and DISC, a small portion of SCC, assortative mixed.
Blogosphere	Power-law degree distribution (λ_in_ = 1.6, λ_out_ = 1.9 [26], λ_in_ = 2.12∼2.38 [8], λ_in_ = 2.15, λ_out_ = 2.95), λ_in_ = 1.34, λ_out_ = 2.6 [17], small world [8], [9], [26], bow-tie structure with a huge SCC [53].
Wikipedia community	Power-law degree distribution (λ_in_ = 2.15, λ_out_ = 2.57), small world [34], bow-tie structure with a huge SCC [34].
Question & answering community	Power-law degree distribution (λ_in_ = 1.87), existence of a large number of hubs [14], bow-tie structure with a huge IN [14].
Twitter community	Power-law degree distribution (λ_in_ = 2.4, λ_out_ = 2.4) [12], a small portion of SCC [54].
Social networking sites	Power-law degree distribution (λ = 2.12), small world, assortative mixed [17].

## Results

In our dataset, there are 11 platforms that participated in the 98 HFS episodes, as shown in Table 1. Figure 2 shows the corresponding HFS network. Table 2 summarizes the network topological properties of the HFS group. In general the network is sparse, as reflected by the small network density and average clustering coefficient values, which indicate a loose organization of HFS groups. This is consistent with our assumption that the HFS organization is inclusive. We observe that the HFS group network had a giant component, which consists over one half of the whole network. Most of the nodes in this giant component are *tianya* users (red). *tianya* is well-known as one of the two biggest HFS platforms (the other one is *mop*, the green nodes in the network). The giant component includes nodes of different colors, indicating the collaborations among different platforms. It is worth noting that one user could have multiple IDs within one platform and/or across different platforms; and not all citations, especially cross-platform citations followed a standard format that can be identified. Therefore, the real cross-platform collaboration frequency should be higher than what the analysis revealed.

**Figure 5:**
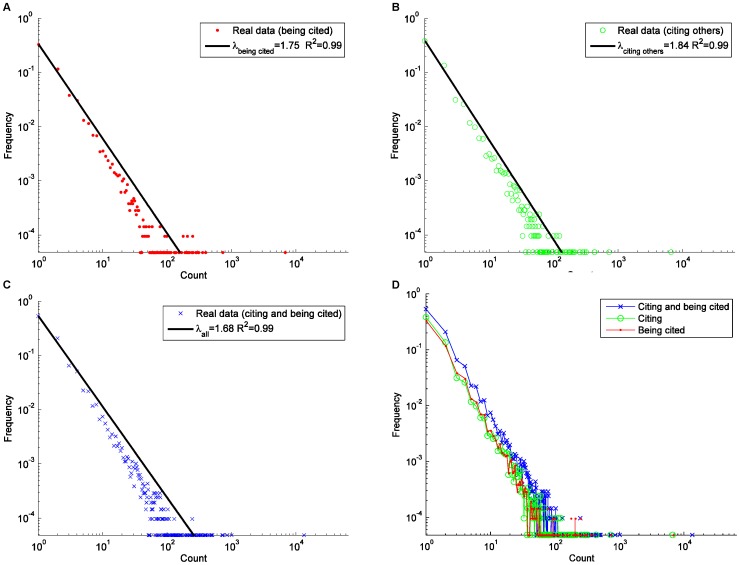
The distributions of the number of being cited and citing others. (A) being cited; (B) citing others; (C) citing and being cited; (D) all.

The second largest component is mainly consisted of *xitek* users, who are mostly photography fans and dedicated a lot of their expertise to the search tasks involving the identification and analysis of photos. Most of the nodes in the third and fourth largest components are *mop* users (green). Since the *mop* forum was changing constantly and not all discussion threads were accessible to non-*mop* users or even low-level *mop* users, the actual number of *mop* nodes and edges could be much larger than what the data indicated. The fact that most of the nodes in the three biggest components were *tianya* and *mop* users revealed that these two nationwide online forums were the two most influential platforms in the HFS group.

**Figure 6:**
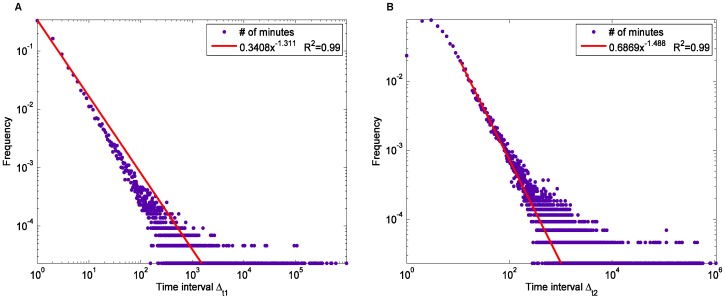
The distribution of time intervals. (A) time intervals Δ_t1_ between two consecutive citations in one discussion thread; (B) time intervals between two linked posts Δ_t2_.

### Bow-Tie Structure

To analyze its social structure, we employed the bow-tie model to study the HFS group. In the bow-tie model, SCC represents the biggest strongly connected component, which is the core of the network; IN represents the component which contains users only cited others’ posts; OUT represents the component which contains users who were only cited by others; TENDRIL and TUBE represent the components that either connect IN or OUT, or both of them, but not connected to SCC; the DISC is the isolated components [32].

**Figure 7:**
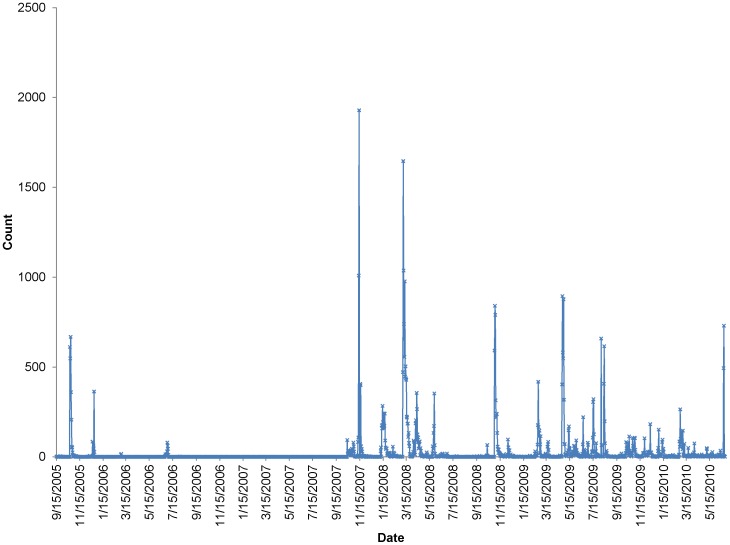
The temporal fluctuations of the citations from 2005 to 2010.

**Figure 8:**
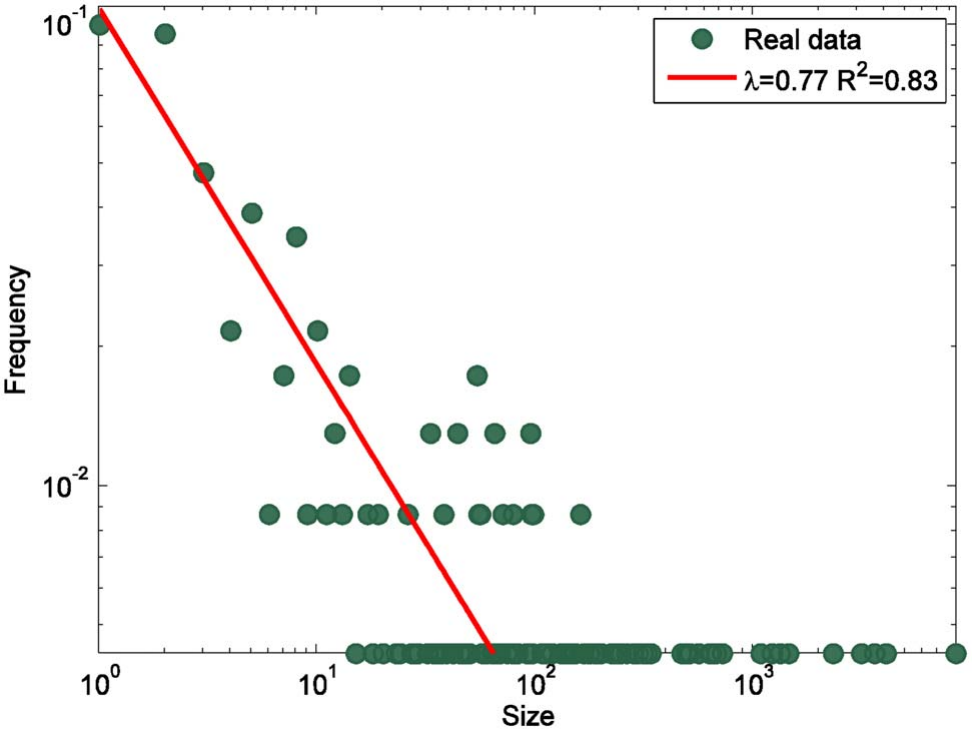
The distribution of avalanche sizes in the HFS group.

### Connectivity and Hierarchical Structure

Table 3 and Figure 3 describe the bow-tie structure analysis. We observe that unlike the World Wide Web, Wikipedia community, Twitter community, blogosphere, as well as Q&A online forum, the HFS group is unique in that it has a smaller SCC and huge TENDRIL (the portion of TENDRIL is similar to the Web. But 44.4% of the nodes are in the disconnected components). This observation indicates that the size of core investigators is small in the HFS group even after we exclude casual nodes. In addition to the core SCC part, the collaboration of the HFS group is also dependent on the existence of a large number of TENDRIL nodes, who help spread and aggregate the information produced by different discussion groups and sub-groups.

**Figure 9:**
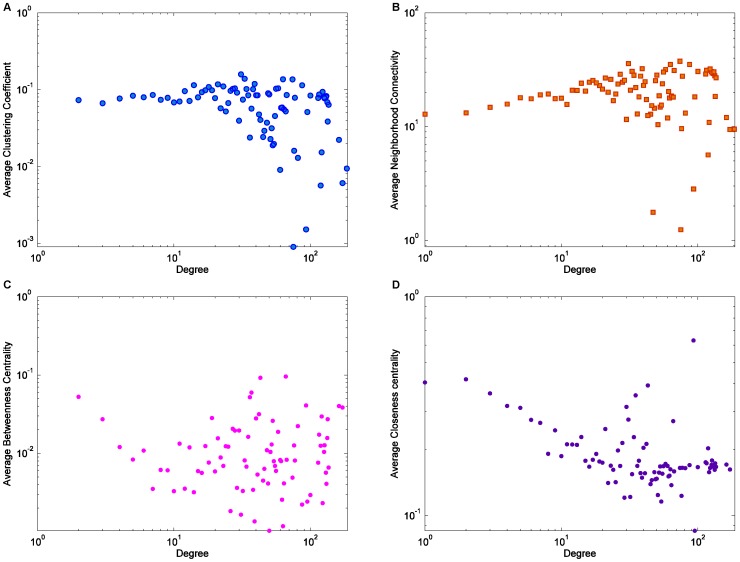
The relationship of the four topological properties and degree. (A) average clustering coefficient; (B) average neighborhood connectivity; (C) closeness centrality; (D) betweenness centrality.

### Degree Distribution

The average degree <d> of the HFS group is much smaller than blogsphere, Twitter, and many other online communities [1], [8], [12], [33], indicating the HFS group is a relatively loose organization. In the HFS group, the in-degree of a node is the number of citations received by this node and the out-degree represents the number of citations generated by the node. As shown in Figure 4, both the in-degree and out-degree distributions of the HFS group network follow a power-law distribution, with similar slope values (λin = 2.07 and λout = 2.20) with R^2^ larger than 0.998 (the algorithm used in this article to fit the power-law function is the Trust Region algorithm). This means that a small number of HFS participants generated most of the citations and only a few HFS participants received most of the citations. Note that the HFS slope values are comparable to those of certain datasets of blogs [26] and question & answering group [14], lower than those of other datasets of blogosphere [8], [9], Wikipedia [34], the out-degree distribution SNS [17], and Twitter [12] (see Table 4), but higher than the in-degree distribution of SNS [17].

**Table 5:** Key HFS participants according to centrality measures.

Rank	ID	In-degree	ID	Out-degree	ID	Betweenness
1	9258	185	12935	145	10	0.014233
2	4389	161	10084	120	12935	0.01241
3	9702	119	10247	117	4389	0.011885
4	1856	118	10081	112	1856	0.011121
5	7110	118	10093	105	12562	0.009119
6	10057	113	2069	102	4009	0.008039
7	16879	95	10265	95	3635	0.007389
8	10184	92	5492	92	3448	0.006876
9	7082	87	10269	91	1923	0.006764
10	5492	83	11440	88	3773	0.006569

### Citation Activities

In order to understand the HFS participants’ citation/reply activities, we show the distributions of the times of an HFS participant’s posts being cited by others and the times of HFS participants citing/replying to other participants’ posts in Figure 5.A and Figure 5.B, respectively. We also present the distribution of times of HFS participants citing and being cited in Figure 5.C and compare the slopes of these three distributions in Figure 5.D. All distributions are power-law type, with a slope ranging from 1.68 to 1.84, meaning that while a few number of participants collaborated with each other actively, many more were not highly involved. This finding is consistent with most existing studies on the collaboration and information spread activities of people in social networks [9], [35], [36]. The power-law distributions observed in the citation activities indicate that in the HFS group, most participants only replied to or were replied by a small number of other participants, and a small number of participants either replied to or were replied by many others.

Moreover, we studied the distribution of Δ_t1_, the time intervals between two consecutive citations in one discussion thread, and the distribution of Δ_t2_, the time intervals between two linked posts (the post being cited and other posts citing it), as shown in Figure 6. The time unit used in this analysis was one minute. The distribution of Δ_t1_ closely follow a power-law distribution with a power of 1.31, indicating that most citations were posted within a short period of time after the previous citations were posted within the same discussion thread. Although the distribution of Δ_t2_ has the highest frequency at Δ_t2_ = 2, it also follow a power-law distribution when Δ_t2_>2, with a power of 1.49, showing that most HFS participants generated links to others’ posts shortly after the others’ posts were posted. The existence of the long tails in both distributions indicates that (a) the discussions could be reactivated after they became less popular; and (b) there were also a number of posts replied by others after a long period of time.

**Table 6:** Key HFS participants according to PageRank and HITS.

Rank	ID	PageRank	ID	Authority	ID	Hub
1	14857	0.003871	9258	0.00436	4389	0.004126
2	4389	0.00358	4389	0.003798	10057	0.003832
3	7082	0.003378	9702	0.002813	10184	0.003242
4	9258	0.00296	7110	0.00279	1856	0.003058
5	9059	0.002245	1856	0.00279	11440	0.003021
6	7110	0.002171	10057	0.002673	5492	0.002874
7	1856	0.002137	16879	0.002251	12935	0.002874
8	9067	0.002094	10184	0.00218	10081	0.002542
9	11567	0.002081	7082	0.002063	2069	0.002432
10	16879	0.001999	5492	0.001969	10265	0.002284

The temporal fluctuations of the citations are shown in Figure 7, with a day as the time unit for analysis. We observe that a series of citation avalanches occurred. This phenomenon is indicative of bursting events as in the self-organized dynamical systems [11], [37]. To validate this hypothesis, we first define an avalanche as a sequence of citations/replies in one discussion thread triggered by the original information posted by the initiator. Thus the number of citations occurred in one discussion thread is the size of the corresponding avalanche. The distribution of the avalanche sizes is shown in Figure 8. We observe that it roughly follow a power-law distribution (λ = 0.77, R^2^ = 0.83), which is similar to the findings in blogosphere [11], indicating the self-organized dynamics in the HFS group.

**Figure 10:**
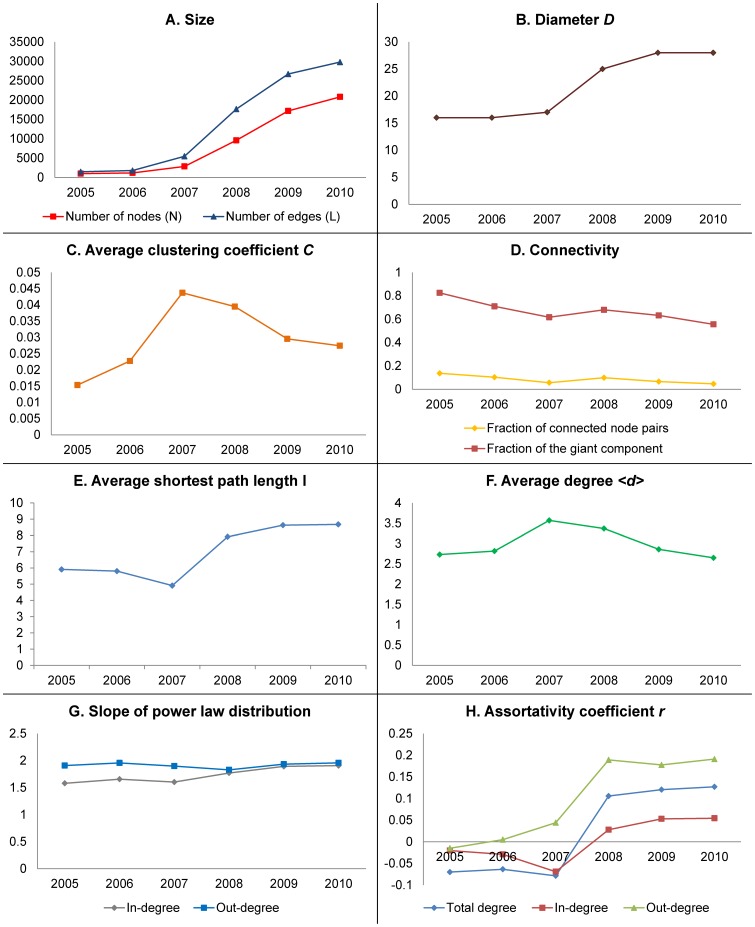
The evolution of the topological properties of the HFS group from 2005 to 2010. (A) the number of nodes and edges; (B) the diameter; (C) the average clustering coefficient; (C) the connectivity features; (D) average shortest path length of all connected node pairs; (E) the average degree; (F) the slope of the power-law degree distribution; (G) assortativity coefficient.

The average shortest path length *l* for all connected node pairs in the HFS group network is 8.679, with a diameter *D* of 28. Both numbers are very small compared to the total number of nodes in the network–20813. In addition, the average clustering coefficient of the HFS group network is 0.027, many times larger than the theoretical prediction for random networks with the same size–0.000069, indicating that the nodes in the HFS group tend to form closed triplets. These observations have shown that the HFS group possesses the small-world property. Furthermore, we observe that only 4% of the node pairs in the network are reachable, which is much lower than the 12% for blogs [8] and 25% for the Web [32]. This finding could lead to the conclusion that even with the small-world property, the information flow in the HFS group is still not easy and highly relied on a small portion of key nodes. However, since most HFS collaboration activities were conducted on the online forums, whose content was open to the public, the information spread did not necessarily have to be conveyed by citations. In addition, traditional media reports also played important roles in publicizing the information. Therefore we still conclude that the information flow in the HFS groups is effective.

**Table 7:** Network analysis of different platforms of HFS group.

Measure	*163*	*baidu*	*dahe*	*fengniao*	*mop*	*sina*	*supervr*	*tianya*	*tiexue*	*xitek*
*N*	125	1240	153	54	1580	171	123	16706	193	465
*L*	112	950	164	36	1413	445	287	25396	144	823
Δ	0.014	0.001	0.014	0.025	0.001	0.031	0.038	0.000	0.008	0.008
*NC*	18	389	15	20	282	3	6	2017	51	26
*N_G_* (%)	85(68.0%)	143(11.5%)	113(73.9%)	18(33.3%)	368(23.4%)	167(97.7%)	114(92.7%)	11524(69.0%)	36(18.7%)	414(89.0%)
*<d>*	1.792	1.436	2.026	1.259	1.797	4.807	4.195	2.802	1.482	3.131
*C*	0.037	0.009	0.015	0.000	0.034	0.136	0.093	0.027	0.000	0.037
*l*	1.105	2.651	3.331	1.586	2.604	2.976	3.297	8.697	1.429	5.152
*D*	3	6	9	2	9	7	7	28	3	17
*λ_in_*	N/A	2.496	1.583	N/A	N/A	1.171	N/A	1.870	N/A	1.750
*λ_out_*	N/A	N/A	N/A	N/A	N/A	1.142	N/A	1.898	N/A	1.772

The existence of hierarchical structures, indicated by the decreasing trend of clustering coefficient with degree, has been widely reported in many real-life networks including social networks, biological networks, the semantic Web, the Internet, among others [38], [39], [40]. However, the HFS group shows a markedly different pattern. The relationship between the average clustering coefficient and the degree (in and out) is shown in Figure 9.A. We observe that when the degree is less than 20, the clustering coefficient is largely independent of the degree. When the degree is larger than 20 (i.e., huge hubs), the distribution of the clustering coefficient becomes fluctuated and scattered without a clear trend, indicating that the hubs in the HFS group are heterogeneous in terms of their hierarchical positions at the mesoscopic scale [19], [41], which will be discussed in the following sub-section. We hypothesize that this characteristic is partially responsible for the diversity of sub-groups as participants can be clustered around very different hubs.

### Heterogeneity and Decentralization

In order to better understand the heterogeneity of HFS participants, we further studied the assortativity of the HFS group network, which is the preference for a participant to collaborate with the others of similar degree (in and out) [42], [43]. The total degree assortativity coefficient *r* for HFS group is 0.127. The in-degree assortativity coefficient *r_in_* is 0.054. The out-degree assortativity coefficient *r_out_* is 0.191. These findings indicate that HFS participants are gregarious, tending to connect to others with similar total degree, in-degree, or out-degree. In particular, the participants are more gregarious in the activities of citing others’ posts (out-degree). As a whole network, the HFS group has the assortative mixing feature, in agreements with the findings in previous research on social networks. The degree assortativity coefficient *r, r_in_, and r_out_* for HFS groups is larger than for certain SNS (MySpace and Cyworld) [18], and Renren [17], but lower than or close to other SNS (Testimonial and orkkut), [18], scientific coauthorship networks, and film actor collaborations [42].

**Table 8:** Types of HFS episodes.

Type	Type ID
*Anti-animal abuses*	1
*Controversial netizens*	2
*Controversial postings on the Web*	3
*Disclosing other ethical issues*	4
*Disclosing unethical or improper acts in public areas*	5
*Discussing doubts about government claims and PR*	6
*Finding product defects and false claims*	7
*Helping with anti-corruption efforts*	8
*Identifying academic ethics and plagiarism*	9
*Inappropriate exposure*	10
*Inappropriate sexual relationship or behavior*	11
*Interesting and unconventional people or events*	12
*Mystery good-looking people*	13
*Other truth-finding tasks*	14
*Political opinions and politicians*	15
*Public safety*	16
*Public services*	17
*Rumors concerning celebrities*	18
*Showing off wealth*	19
*Traffic accidents*	20

**Table 9:** Network analysis of different types of HFS sub-groups (for the slope of power-law distribution correlation, we used “N/A” to indicate that the corresponding R^2^ value is less than 0.8).

Measure	1	2	3	4	5	6	7	8	9	10
*N*	187	1540	625	312	659	1556	1758	2607	207	797
*L*	324	1145	655	194	468	1396	5198	3425	198	643
Δ	0.019	0.001	0.003	0.004	0.002	0.001	0.003	0.001	0.009	0.002
*NC*	34	492	89	125	216	430	146	370	38	211
*N_G_* (%)	114 (60.96%)	143 (9.29%)	414 (66.24%)	57 (18.27%)	136 (20.64%)	428 (27.51%)	662 (37.66%)	1717 (65.86%)	121 (58.45%)	281 (35.26%)
*<d>*	3.144	1.413	1.9744	1.212	1.363	1.694	5.064	2.552	1.816	1.609
*C*	0.061	0.006	0.007	0.001	0.004	0.017	0.106	0.012	0.023	0.025
*l*	3.281	2.579	4.089	2.104	2.195	3.943	3.529	5.142	2.624	2.806
*D*	7	6	10	5	6	11	17	14	5	7
*λ_in_*	N/A	2.087	2.222	N/A	1.643	1.63	1.526	1.629	1.761	N/A
*λ_out_*	N/A	N/A	1.878	2.447	N/A	1.914	1.339	1.824	N/A	3.128
Measure	11	12	13	14	15	16	17	18	19	20
*N*	2499	110	901	462	1227	812	976	695	1437	1430
*L*	4654	64	643	430	1093	741	1421	747	2808	1058
Δ	0.001	0.011	0.002	0.004	0.001	0.002	0.003	0.003	0.003	0.001
*NC*	215	47	281	108	278	197	88	107	189	409
*N_G_* (%)	1702(68.11%)	6 (5.45%)	39 (4.33%)	113 (24.46%)	367 (29.91%)	227 (27.96%)	795 (81.45%)	345 (49.64%)	905 (62.98%)	426 (29.79%)
*<d>*	3.513	1.145	1.385	1.775	1.654	1.69	2.697	2.003	3.411	1.462
*C*	0.038	0	0.004	0.014	0.01	0.022	0.015	0.017	0.067	0.001
*l*	5.416	1.123	1.682	3.298	3.426	3.221	5.906	5.248	4.188	2.979
*D*	14	2	6	9	10	8	16	16	15	13
*λ_in_*	1.461	2.691	2.041	2.073	1.986	1.863	1.577	N/A	1.63	1.645
*λ_out_*	1.78	4.907	3.335	N/A	N/A	N/A	1.932	1.946	1.404	1.749

**Figure 11:**
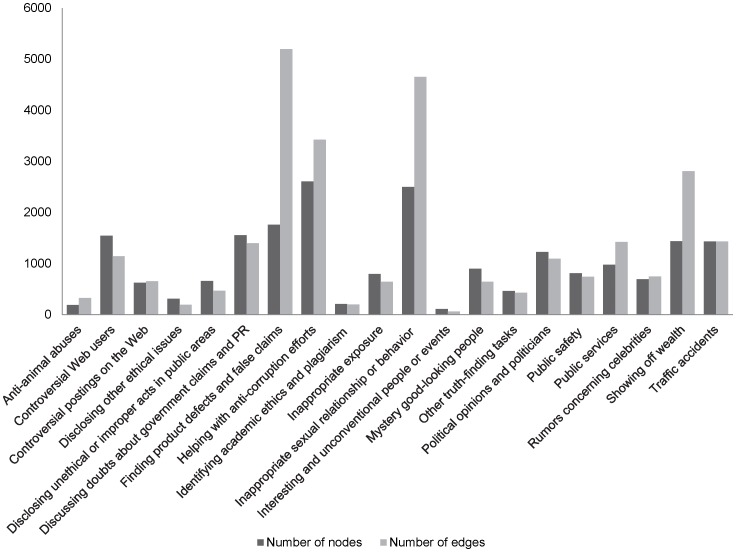
The size of sub-groups of different types.

In Figure 9.B, we illustrate the relationship between the average neighborhood connectivity and the degree. The average neighborhood connectivity of a node is defined as the average of the number of neighbors of this node’s neighbors. For the nodes with degree lower than 20, the increasing trend reinforces the observation that the HFS group is assortative mixed for nodes with a low degree. However, we find that the distribution becomes more and more fluctuated and scattered as the degree increases, similar to that of the average clustering coefficient. This indicates that the HFS group is assortative for some key participants, but disassortative mixed for other key participants. In other words, the key participants are heterogeneous in terms of the assortative mixing patterns.

We have also studied the relationship among closeness centrality, betweenness centrality [44], and degree, as shown in Figure 9.C and Figure 9.D. In most other social networks, both the closeness centrality and betweenness centrality are positively correlated to degree [40], [45]. However, for HFS, both the closeness centrality and betweenness centrality are negatively correlated to degree when the degree is less than 20. Similar to Figure 9.A and 9.B, the distributions of closeness and betweenness become fluctuated and scattered when degree exceeds 20. The decreasing trend of closeness centrality shows that the HFS participants choose to connect to key participants without reducing the distance between them to others. In addition, the decreasing trend of betweenness centrality demonstrates that the HFS group is a distributed network, with no single hub controlling most of the information diffusion paths. The scattered points in the distribution of average clustering coefficient, neighborhood connectivity, betweenness centrality, and closeness centrality for degree larger than 20 (see Figure 9) shows the heterogeneity and complexity of the network structure at the mesoscopic scale [19], [41]. This pattern might be the result of the occurrence of the sub-groups structure, which has not been fully analyzed here and needs further investigation. We have also studied the relationships between the four topological properties and both in-degree and out-degree. We found that these relationships manifest the same patterns as those presented above based on the total degree.

The study of the heterogeneity and decentralization helped us understand the organization of HFS from another angle: the key HFS participants, which were represented by the hubs with a degree larger than 20, had very different collaboration patterns, showing that the HFS participants were *decentralized*. In addition, since the key participants did not always tend to collaborate with others with similar attributes, the *diversity of opinions* and *independence* of different key participants could be maintained in HFS groups, which are also keys to the success of the search task, according to the criteria to characterize the wisdom of crowds proposed in [46].

Table 4 summarizes and compares the major findings of the HFS group and other online communities.

### Key HFS Participants

It is important to find the key contributors, spreaders, and transmitters in the HFS group studies. One of the most common measure is the degree centrality [44]. In the aggregated HFS group network, nodes with high in-degree represent the participants that received lots of citations from other participants (key information contributors); nodes with high out-degree represent the participants that generated many citations to participants (key information carriers). Betweenness centrality is another popular measure to find key information transmitters [44]. Nodes with high betweenness centrality are the participants that occurred on many shortest paths between other pairs of participants in the group. Table 5 shows the ranking according to degree and betweenness centralities. To avoid privacy issues, we replaced the real IDs with unique digital IDs for all nodes.

PageRank and Hyperlink-Induced Topic Search (HITS) are two prominent ranking algorithms for social network analysis [47]. A node in the HFS group network has high PageRank score if it is linked by many nodes with high PageRank score [48]. HITS, as a precursor to PageRank, could identify hub nodes and authoritative nodes in networks. The hub score and authority scores are dependent on the node’s in-degree and out-degree, respectively. In the HFS group network, a node with a high hub score is a participant who has provided valuable information for many other participants, and a node with a high authority score is a participant that has obtained knowledge from many good hubs [49]. The top ten highest scoring HFS participants according to PageRank and HITS metrics are listed in Table 6.

Comparing two pairs of rankings: in-degree vs. out-degree and hub score vs. authority score, we observe that there are few overlaps. It could be observed that most of the key information transmitters unveiled by the ranking of betweenness centrality are not key information contributors and carriers (except 4389 and 1856), which implies the complexity of the HFS group at the mesoscopic level [19], [41]. This finding shows that the key HFS information contributors, information carriers, and information transmitter are from three different groups of HFS participants and few participants play more than one significant roles in HFS. It also echoes the heterogeneity of key HFS participants observed in previous sections.

### Evolution of HSF Group

To understand the evolution of citation activities over the time span in our dataset (2005–2010), we analyzed (a) the changes of the size, (b) diameter, (c) average clustering coefficient, (d) connectivity features, including the fraction of connected node pairs and the fraction of the giant component, (e) average shortest path length of connected node pairs, (f) the average degree, (g) the slope of the power-law in-degree and out-degree distributions, and (h) the assortativity coefficient of total degree, in-degree, and out-degree, as shown in Figure 10.

We observe that there are clear changes of all measures in the year of 2008. There are several reasons for these changes. First, the number of HFS episodes each year has been steadily growing since its inception [1]. Second, there were several major events taking place in 2008, including the Beijing Olympic Games and the Sichuan Earthquake. As a result, there were an extraordinary number of episodes about public services and safety in this year.

Many social networks have been identified as having a decreasing diameter while the size of the network is increasing [50]. But as we can see in Figure 10.B, the diameter grew slowly from 2005 to 2007, and from 2008 to 2010, but it experienced a major jump in 2008. It has also been found that many real world social and technological networks follow a densification law, which means that the number of edges in social networks grows superlinearly in the number of the nodes over time: E(t) ∝ L(t)^α^ with α ranging between 1 to 2 [8], [50]. A previous case study of HFS also unveiled that that small HFS network for a single episode followed this densification law for a time window of two months, with α = 1.21 [1]. Surprisingly, in this study we observe that the evolution of the whole HFS group does not follow the densification law, as although the data followed the superlinear function, the power α is smaller than 1 (α = 0.83, R^2^ = 0.99). These two phenomena show that the HFS group is becoming increasingly dispersed, which indicates that HFS participants tended to form more distributed collaboration groups. However, not obeying the densification law does not necessarily indicate that information transmission is blocked in the network, since both the traditional and social media would collect and publish the important findings of small collaboration groups.

### Comparison of Different Platforms

As described in the Data subsection, there were 11 platforms involved in the 98 episodes in the dataset. Although it was found that there were a small number of Web users participating HFS in multiple platforms, performing as the information bridges [1], the organization of HFS group was still very loose, as shown in the previous sections. Participants from different platforms are largely isolated into different disconnected components, as shown in Figure 2. For instance, over 95% of the nodes in the giant component are made up of users from *tianya*; the second largest connected component was mainly consisted of *xitek* users; most of the nodes in the second and third largest connected components are from *mop*. In fact, all the connected components with more than 20 nodes consist of users mainly from a single platform. Therefore, to better understand the collaboration patterns of HFS participants on each platform, we split the aggregated the HFS group network into 10 HFS sub-groups, each of which only contained the participants and their relationships in one platform (the network of *moveshow* was excluded because of its very small size, since most discussion threads on *moveshow* were inaccessible [23]). Table 7 summarizes the analysis of each network. Because the user IDs shown in news comments on *sina* are highly aggregated–if a user did not provide an ID, she or he will be labeled by the location information according to the her or his IP address (for example, “user from Beijing” and “user from America”), the numbers of nodes and edges are much smaller than they supposed to be. The network of *sina* is also denser than it should be due to the data restriction. Platform *163* had a similar aggregated labeling scheme. However, since the platform *163* could display partial IP addresses of a user, the overlapping problem is not as serious as it for *sina*.

The analysis reveals that the total number of collaborators and citations involved in general nationwide platforms is much larger than the local platforms (whose users were mainly local residents) and the forums specialized for professional users (fans of photography, pets, military, etc.). However, the networks of local and professional platforms are much denser than the nationwide and general ones, as shown by their higher network densities and average clustering coefficients. For example, the network density is 0.001 for *mop* and *baidu*, and nearly 0.000 for *tianya*. In contrast, the network density for *supervr* and *xitek* is 0.038 and 0.008, respectively. The average clustering coefficient for *mop*, *baidu*, and *tianya* is 0.034, 0.009, and 0.027, respectively. They are all below the values for *supervr* and *xitek*–0.093 and 0.037, respectively, though the gap is smaller. These observations imply that although the sizes of local and professional users are smaller, there are more collaboration occurred among them. In fact, according to our dataset, most offline investigation activities were initiated and organized by participants of local and professional platforms. This is of no surprise because (a) the population of Web users in local and professional platforms is smaller; (b) the information in nationwide and general platforms is broad and comprehensive, thus attracting more Web users to participate the discussion; and (c) members of local and professional platforms are more interested in certain topics that are relevant to their benefits and interests. The episodes that attracted local and professional users often required local investigations, or specialized knowledge in a certain field. Therefore, if the topic of an HFS episode was what they were interested, they were more likely to participant and discuss with other fellows. Once they were involved in an HFS episode, they always played significant roles. For instance, in the *South China Tiger* episode, *xitek* users employed their knowledge in photography to provide convincing evidence to prove that the photo of the tiger was a fake [1]. In another case, *the Neihu cat-abuse* episode, most HFS activities were conducted by users from forums of pet lovers [23]. A third example is the *outrageous hair-cut* episode, which happened locally. Most discussions of this episode were among local citizens [1].

Our findings are contrary to the previous study of co-location for scientific innovation. In scientific research, international collaboration usually demonstrated higher research level than domestic and local collaboration in various disciplines [51], [52]. However, in the HFS group Web users of local forums collaborate more and show higher level of investigation than nationwide collaboration. This phenomenon is due to the fact that the HFS group has a stronger purposive incentive because participants of local and professional platforms have more relevant knowledge and higher interests in the topic. According to theories of social organizations, stronger purposive incentive is necessary to ensure participants to dedicate time into HFS and maintain loyalty to the HFS [31]. Therefore, the HFS participants are able to (and more likely to) remain interested in HFS episodes and conduct real-world investigations (for local cases).

### Comparison of Different Types of HFS Episodes

In this subsection, we present the analysis of different HFS sub-groups identified by different types of HFS episodes, as summarized in Table 8 and Table 9. For more details of the classification, please refer to [2], [6]. We did not study the type of “*net mobs*” because there was few data of this type available online due to the fact that many discussion threads of this type have been deleted. Figure 11 shows the size of the sub-groups of different types.

We observe that the networks of episodes that require certain degree of professional knowledge and episodes that involve professional knowledge background and/or ethical issues are much denser, indicating that there are more collaboration occurred during HFS episodes of these types. For example, the network density for “*anti-animal abuses*” and “*identifying academic ethics and plagiarism*” is 0.019 and 0.009, respectively. The average clustering coefficient for networks of these two types is 0.061 and 0.023, respectively. These values are larger than most of other types of sub-groups. Similar to the above discussion, this phenomenon is due to the fact that users involved in these types of episodes shared common interest and had similar background related to the episodes. They were also more motivated when the HFS episodes were related to their own backgrounds, benefits, and interests. Thus they were more likely to contribute their own knowledge and collaborate with each other. The episodes involved of ethical issues also motivated HFS participants to collaborate and conduct investigations. On the other hand, for episodes that did not require much professional knowledge, the networks were sparser. There is no surprise for this since for general episodes that did not involve professional knowledge or ethical issues, a large portion of participants treated HFS as an entertainment and did not pay much attention or contributed valuable information. As a result, most of posts produced by this group of users had neither cited others’ posts nor received citations from others.

In addition, we find that the largest sub-group is the participant network for “*helping with anti-corruption efforts*,” the third largest network is for the type of “*finding product defects and false claims*.” This finding echoes our previous findings that a large portion of HFS episodes have played positive roles in the society [1].

## Discussion

In this research, we have analyzed the most comprehensive HFS group so far that involved 98 typical HFS episodes. We find that similar to other online social networks, the HFS group possesses the scale-free and small-world properties. However, the HFS group network is sparser and less centralized than other online groups and communities. We demonstrate that the unique features of decentralization and diversity of the HFS group lead to the key of its success. In addition, the evolution of the HFS group show that it has been becoming increasingly dispersed since its inception. It is observed that the collaboration patterns heavily rely on a small number of key players. Rankings of key HFS participants according to different measures show that the key information contributors, carriers, and transmitters of different roles belong to different groups of HFS participants and there are few participants that played more than one significant roles in HFS.

To better understand the collaboration patterns within the HFS group, we further split the aggregated HFS group into two sets of sub-groups according to the platforms that nodes belonged to and the types of the HFS episodes, respectively. The network analysis of both sets demonstrate that (a) the sizes of the HFS sub-groups on nationwide platforms are larger than professional and local ones; (b) the collaboration among the HFS participants from nationwide platforms occurred less frequent than the collaboration from local and professional platforms; and (c) collaboration in episodes that involved certain degree of professional knowledge or ethical issues was more frequent than that in episodes with a general topics without specific knowledge requirement or ethical issues.

HFS has been ubiquitously integrated into people’s everyday lives in China. HFS, as a type of crowdsourcing and cyber-enabled social movement, could provide rich data sources for many data-driven research and testing social theories and hypotheses. In future work, we will focus on the automatic detection and tracking of HFS episodes and the modeling of dynamic information structure in HFS groups to understand how the context and social roles affect the behaviors of HFS participants. Clearly, more research on topological characteristics, collaboration patterns, and information aggregation of HFS groups are needed from the perspective of sociological and psychological studies.
